# Some rare presentations of hydatid cysts: two case reports

**DOI:** 10.1186/1757-1626-2-62

**Published:** 2009-01-18

**Authors:** Syed H Zaidi

**Affiliations:** 1 Department of Surgery, PNS Rahat Hospital, Karsaz, Karachi, Pakistan

## Abstract

Hydatid disease is a considerable health problem worldwide. Two case reports of relatively uncommon presentations of the disease are presented.

The first case is that of a 25 years old female from region of Afghanistan that borders Pakistan's Baluchistan province. She presented with cough, hemoptysis and left hypochondrium pain due to concurrent involvement of the right lung and the spleen due to hydatid disease, whilst sparing the liver.

The second case is that of a 32 years male from the same region of Afghanistan as above. He presented with upper abdominal discomfort, postprandial vomiting and jaundice due to a hydatid cyst involving the head of the pancreas only.

## Background

Hydatid disease, caused by the larval stage of the parasite Echinococcus, is a considerable health problem worldwide. E. granulosus accounts for the majority of the cases whilst *E multilocularis *and *E vogeli *are rare. Humans happen to be accidental or incidental intermediate host and, as far as the parasite is concerned, a dead end. Hydatid disease can involve any organ. The liver is the most common organ involved and, together with the lungs accounts for 90% of the cases. Other sites of involvement are muscles (5%), bones (3%), kidneys (2%), brain (1%), and spleen (1%) [1]. Pancreas is affected in 0.25–0.75% of adult cases, the mode of infestation being hematogenous, via pancreatic or bile duct as well as lymphatic [2].

This paper emphasizes the fact that hydatid disease should be suspected in cystic lesions affecting any organ in the body, especially in endemic areas of the world.

## Case 1

**Figure 1:**
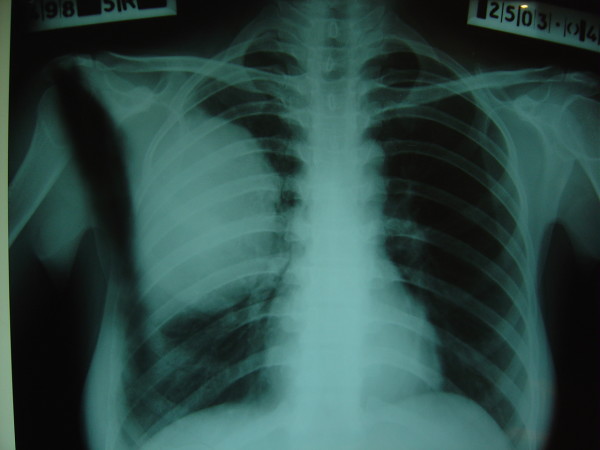
**Case 1 – Hydatid cyst in the right lung**. Posteroanterior chest film shows a well demarcated oval opacity occupying the right middle zone.

**Figure 2:**
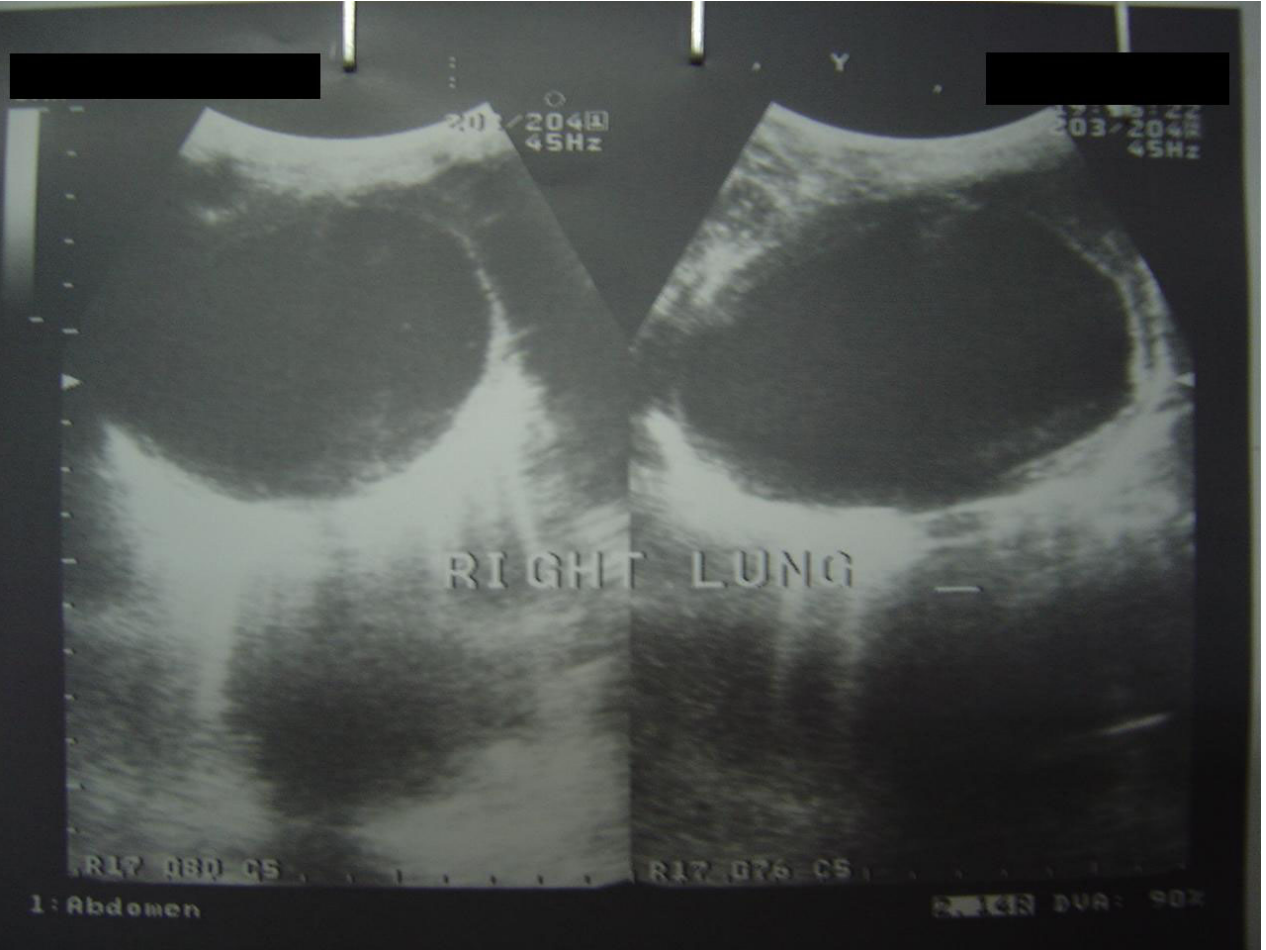
**Case 1 – Ultrasound of right lung shows a large cystic lesion with a with the double wall sign**.

**Figure 3:**
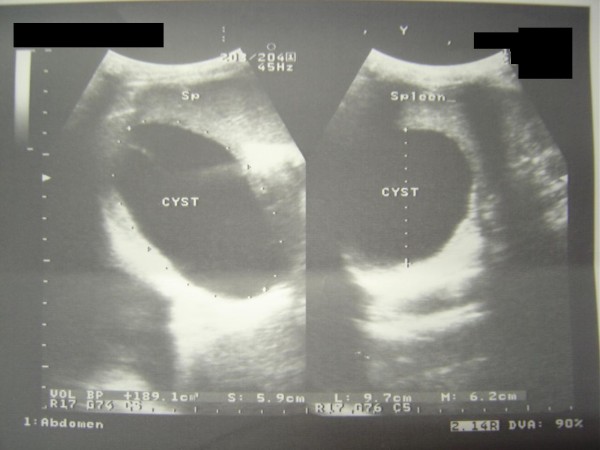
**Case 1 – Hydatid cyst of the spleen**. Ultrasound of the spleen showing a cystic lesion in the spleen.

**Figure 4:**
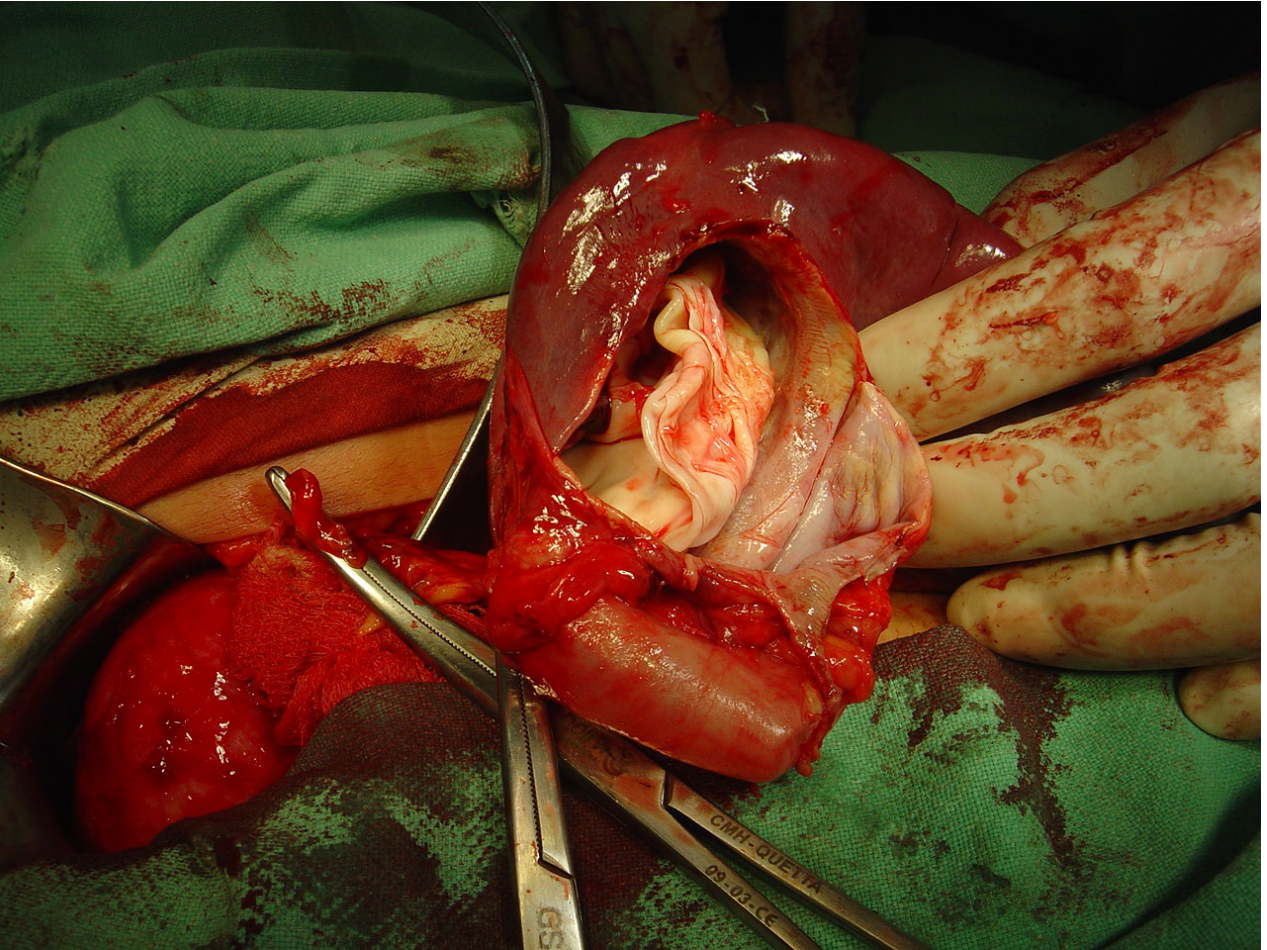
**Case 1 – Operative view Hydatid cyst of the spleen**. The cyst is being removed whilst preserving the spleen.

A 25 years old female from Afghanistan presented to the medical out patient department of Combined Military Hospital Quetta with complaints of cough for 2 years, and hemoptysis and left hypochondrium pain for the last six months. There was history of occasional fever but no weight loss. Her weight was 48 Kg. Breath sounds were diminished over right mid zone. She had received various medications including anti-tuberculous treatment in Afghanistan. Posteroanterior and right lateral chest x-rays (Fig 1) revealed a large dense round well demarcated opacity involving the mid zone of the right lung field posteriorly. Ultrasonography revealed large simple cysts in the right lung (Fig 2) and the spleen (Fig 3). Both cysts showed the classic double wall sign. The liver and the rest of the abdomen were free of cysts. Hemoglobin, total leukocyte count and differentials were normal. A large unilocular hydatid cyst was removed form the right lung through a posterolateral thoracotomy. Oral albendazole 400 mg b.i.d. was given for 5 days preoperatively and 4 weeks postoperatively. The splenic cyst (Fig 4) was removed six weeks later, preserving the spleen. The patient's immediate recovery was remarkable and her symptoms were relieved completely.

## Case 2

**Figure 5:**
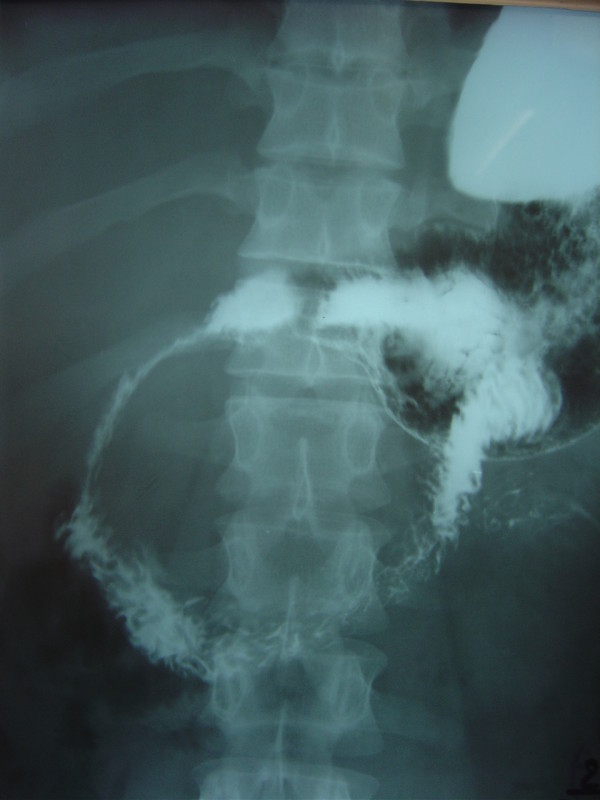
**Case 2 – Hydatid cyst head of pancreas-upper GI contrast using barium shows pressure effect of the cyst on the duodenum**.

A 32 years male from Afghanistan presented to the surgical out patient department of Combined Military Hospital Quetta with complaint of upper abdominal discomfort and postprandial vomiting of 3 months duration. He complained of dark coloration of the urine and yellowness of the sclera for the last one month. The frequency of vomiting had increased over the last 2 weeks. He had lost approximately 5 Kg weight in the last one month and presently weighed 62 Kg. Physical examination revealed mild jaundice and an approximately 15 cm mass in the epigastrium. Barium meal was suggestive of a large radiolucent mass involving the head of the pancreas with enlargement of the duodenal curve and pressure effect resulting in partial duodenal obstruction (Fig 5). Ultrasound was suggestive of a large hydatid cyst, containing a few daughter cysts, involving the head of the pancreas. Hydatid serology was positive. Hemoglobin, total leukocyte count and differentials were normal. Liver function tests revealed serum bilirubin of 128 mg/dl, alkaline phosphatase 1248 u/l and ALT 38 u/l. At laparotomy there was a thin pericystic wall anteriorly and the cyst was removed relatively easily. No gross biliary leakage was seen. The region was drained and the abdomen closed. The patient made and uneventful recovery with complete amelioration symptoms, clearing of jaundice and normalization of the LFTs. The serum amylase remained normal and the drain was removed on the 5^th ^postoperative day. The patient was given oral albendazole 400 mg b.i.d for 4 weeks as a prophylactic measure.

## Discussion

The adult worm resides in the intestine of its definitive host, the dog and related carnivores. The eggs passed in the feces are ingested by grazing sheep, goats and cattle. The eggs hatch, penetrate the host's intestinal wall and reach the liver through the portal vein. From there they are distributed by the bloodstream to the lungs and other organ systems. Eggs become transformed to the larval stage, the scolex, which can continue to multiply asexually indefinitely within the hydatid cyst. The natural cycle is completed when a hydatid cyst is devoured by a canine host. The multiplication of the larval scolices results in a slow but steady physical enlargement of the cystic colony. Since the enlargement is very gradual the patient's symptoms are rarely acute. The cyst consists of three layers. The outer most, or the pericyst, is an adventitial layer of host origin. The middle layer is the outer chitinous covering of the parasite or the laminated membrane. The innermost germinal layer gives rise to the scolices [3].

The usual mode of acquiring the infection is through ingestion of contaminated vegetables. Symptoms are caused by pressure effects but are vague initially. Pain, cough, low-grade fever, and the sensation of abdominal fullness are common features. As the cyst grows, the symptoms become more specific depending on the specific structures involved. Secondary complications include of infection or rupture of the cyst [4].

Theoretically no organ is immune form hydatid disease. When the relatively rare sites are involved, the mainstay of diagnosis remains a high index of suspicion supplemented by radiologic and hydatid serology [5]. Although eosinophilia is expected in patients with parasitic infestations, it has been reported to be present in only 25% of cases of hydatid disease [1]. Surgical removal remains the main form of definitive treatment. Chemotherapy is indicated in inoperable cases because of location, multiplicity of organ involvement or in patients with serious medical conditions [1].

## Consent

Unfortunately, the patients could not be traced to obtain written informed consent. I believe that this case report contains a worthwhile clinical lesson that could not be made as effectively in any other way. I expect that a reasonable person would not object to the publication since every effort has been made so that patient remains anonymous.

## Competing interests

This is a single author case report. There are no competing interests.

## Authors' contributions

This is a single author case report. I have personally kept records of my private cases, like the ones included here. Data retrieval, compilation and writing of the manuscript were done by me.
